# Malaria transmission and morbidity patterns in holoendemic areas of Imo River Basin of Nigeria

**DOI:** 10.1186/1756-0500-4-514

**Published:** 2011-11-24

**Authors:** Uchechukwu M Chukwuocha, Ikechukwu NS Dozie

**Affiliations:** 1 Department of Public Health Technology, Federal University of Technology, Owerri, Nigeria

## Abstract

**Background:**

This study determines the relationship between malaria transmission intensity and morbidity in holoendemic areas of Imo River Basin, Nigeria.

**Results:**

Standard entomological and parasitological techniques were used to determine transmission intensity and parasite rates respectively while sociocultural methods and review of hospital records were used to determine morbidity patterns. The average transmission rate was 16.1 infective bites per person per night (ib/p/n). The average malaria specific morbidity rate for the study area was 30.2%. These parameters showed no significant differences among the communities studied (*P *> 0.05). Transmission intensity and morbidity rate had a linear relationship such that high transmission intensity corresponded with high morbidity rate and vice versa.

**Conclusions:**

This therefore puts to rest discrepancies about the relationship between malaria transmission and morbidity in the study area and calls for serious scaling up of the insecticide treated nets strategy especially in high transmission areas and seasons. Concerted efforts should also be made towards production of transmission blocking vaccines.

## Background

The relationship between malaria transmission intensity and overall incidence of the disease has remained complex. Morbidity rates of the disease seem to be insensitive to differences in transmission over a wide range of intermediate values. Efforts towards control of the disease include reduction of human-mosquito contact using insecticide treated nets or materials. This and other methods such as the transmission blocking vaccine depend on reduction in transmission intensity. However, there seems to be a conflict with these strategies because there are concerns that bed nets, by delaying the acquisition of immunity, may merely shift the burden of disease to an older age group, resulting in little or no long-term benefit or potentially detrimental consequences for the incidence of severe disease-the so-called rebound effect [1,2]. There is also evidence that low to intermediate levels of transmission result in higher incidence of severe disease [3,4]. Supporting these evidences, are suggestions that at high transmission intensities exposure to the parasite occurs very early in life, permitting the development of acquired immunity which protects the individual by other mechanisms (e.g. maternal antibodies) from severe disease [4]. On the other hand, data from Ghana, suggest that a tenfold decrease or increase in malaria transmission is associated only with a twofold decrease or increase in malaria morbidity [5]. In that study, number of malaria attacks were compared between Accra (1 infective bite per person per year), Kumasi (20 infective bites per person per year) and Cape coast (200 infective bites per person per year). Despite these major differences in transmission intensity, the cumulative number of malaria attacks by the age of 60 years was pretty similar -30, 62 and 43 respectively. This deduction is corroborated by findings from Nigeria where each 10 fold increase in the Entomological Inoculation Rate (EIR) corresponded with a 1.6 fold increase of incidence of clinical malaria [6]. Quantifying the relationship between transmission levels and the incidence of clinical attacks, it has also been noted that for low levels of transmission i.e. between 0.001 and 0.1 infective bites per person per year, the incidence of malaria attacks is probably directly proportional to the level of transmission in adults as in children [7]. But for higher levels of transmission like 1, 10, 100 and 1000 infective bites per person per year, the data suggests that malaria morbidity in Africa varies, with higher clinical incidences among children than adults [7].

These conflicts create situations where useful policies and effective planning and implementation of strategies for sustainable malaria control become difficult. It therefore becomes paramount to clearly understand how transmission intensity affects morbidity for proper planning, effective implementation and evaluation of interventions.

This study ascertains the relationship between malaria transmission intensity and patterns of morbidity in holoendemic areas of Imo River Basin of Nigeria.

## Methods

### Study area

The study area consists of parts of Imo State, Nigeria located between latitude 5° 10^1 ^and 5° 51^1 ^North, longitudes 6° 35^1 ^and 7^0^28^1 ^East known as the Imo River Basin. It is bordered on the North by Anambara State, on the South and West by Rivers State and on the East by Abia State. The area has a population density of 458 persons/km^2 ^and the majority of the population is broadly dispersed in a vast number of rural settlements. There are two main climate regimes: a dry season and a wet season. The mean annual rainfall is between 1,800 and 2,500 millimeters per year. The maximum and minimum temperatures are 31.9°C and 22.5°C respectively while the daily sunshine rate is about 4.4 h. Average relative humidity is about 74% occurring mostly during the wet season, while the rate of evaporation and evapotranspiration are 3.0 mm/day and 136 mm/month respectively. The vegetation is typically rain forest.

### Criteria for selection

The Area selected for this study includes Ezinihitte, Aboh Mbaise and Ahiazu Mbaise LGAs. The area apart from being a tropical rain forest which supports breeding of mosquito vectors has no efficient water supply system; hence the inhabitants rely on itinerant water vendors and roof catch during raining months for water supply. Water from the different sources is stored in drums, clay pots and all sorts of metal and plastic containers. The use of container storage of roof water increase breeding points for mosquitoes in the raining months while in the dry season, the seasonal streams, drying up pools, puddles and dug tanks become breeding pools for mosquitoes and their ecological associates. These areas experience stable malaria transmission all year round [8]. More importantly, they were being considered to be selected for the national insecticide treated net distribution program which therefore necessitates that this study should be carried out.

### Study design and sampling method

This study was designed to be analytical in nature. Households for the study were selected through a systematic random sampling technique. The technique involved spinning a bottle at the centre of each village which is the market square. The first household in the direction the bottle pointed was picked and assigned a number; every two households were picked until the village was covered. Informed oral consent was also sought and obtained from households that were selected to participate in the study.

### Pre-disease survey logistics

The pre disease survey logistics included visits to Local Government Area (LGA) chairmen, traditional rulers of the selected communities and village heads to explain the purpose of the survey and solicit for co-operation. Part of the pre-disease survey logistics included mobilization of the communities and the selection/training of village based field assistants (VBFA) (Male and Females).

### Entomological study

Indoor resting mosquitoes were caught fortnightly in randomly selected households in each community between 1800-1600 h using the Pyrethrum Spray sheet Collection (PSC) after all occupants and easily movable objects were removed and immovables were covered in each consented household. White sheets were carefully laid on the entire floor by two assistants. All doors and windows were shut and holes and openings eg between hinges were covered with newsprints. The rooms were then space sprayed with pyrethrum spray and kept closed for 10 min after which they were opened. Fallen mosquitoes were collected from the sheets and packed into petri dishes with labels of the house numbers, date of collection and transported to the laboratory for determination of transmission intensity or entomological inoculation rates.

In the laboratory, the female mosquitoes were identified and dissected to determine parity by observing the degree of coiling of their ovarian tracheoles [9]. The salivary glands of parous mosquitoes were extracted, stained with giemsa and examined for malaria parasites under the microscope [10]. Malaria transmission intensity expressed as Mean entomological Inoculation rates (EIRm) were then calculated as infectious bite per person per night (ib/p/n) using standard formula [9].

### Malaria morbidity study

Structured, pretested questionnaires were administered to one mother/caregiver in each of six hundred and ninety nine (699) selected households by trained field based assistants. In addition key informant interviews were also conducted with 60 community health workers to determine the malaria specific signs and symptoms among children in the study area which includes relapsing fever for 48 hours and headache [5]. Records of total number of outpatient attendance attributed to malaria between 2007-2010 from selected health facilities in the study area were also analyzed. Annual morbidity attributable to malaria was calculated as percentage malaria prevalence of total outpatient cases for the study period.

### Ethical approval

Ethical approval for this study was given by the ethical committee of the Federal Medical Center, Owerri Nigeria. The study was also reviewed by the institutional Review Board (IRB) of the Department of Animal and Environmental Biology, Imo State University, Owerri, Imo State.

## Results

Malaria transmission rates were relatively high. The pattern of human entomological inoculation rates (EIRm) for the study communities was almost similar, although lower in communities located at higher altitudes (Aboh - 4.4ib/p/n, Enyiogugu - 8.4ib/p/n, Ibeku-6.1ib/p/n) and Ahiara - 8.4ib/p/n) (*p *< 0.05) (Table 1).

**Table 1:** Transmission rates of malaria in the study area expressed as mean entomological inoculation rates(EIRm) per community

Study Area	Altitude (m)	EIRm (ib/p/n)
Ezinihitte LGA		

Itu	175	22.7

Chokoneze	164	17.0

Akpodim	123	23.4

Ihitte	182	15.0

Onicha	121	21.2

Obizi	121	20.8

Aboh Mbaise LGA		

Aboh	326	4.4

Enyiogugu	403	8.4

Lorji	187	20.9

Ogbor	215	12.2

Uvuru	123	22.3

Ibeku	400	6.1

Ahiazu Mbaise LGA		

Afor Oru	267	18.8

Ahiara	340	8.4

Oparanadim	263	18.9

Ihitte Afoukwu	261	17.0

Umuokirika	263	20.9

Nnarabia	318	12.5

**Figure 1:**
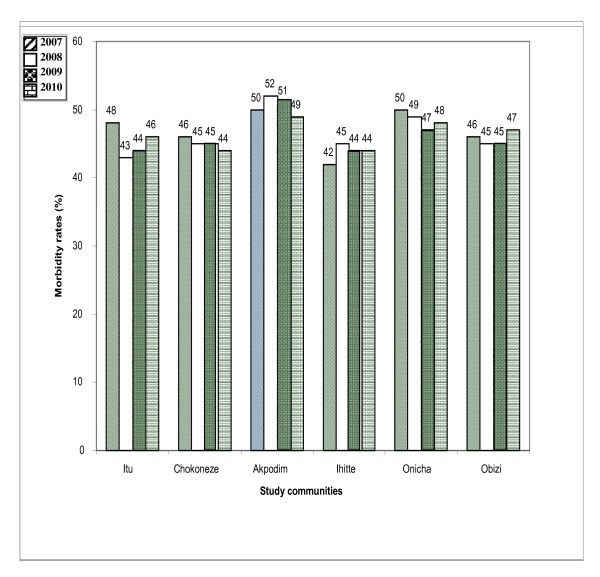
**Malaria morbidity rates as a proportion of total outpatients in health facilities in study communities in Ezinihitte LGA between 2007-2010**.

**Figure 2:**
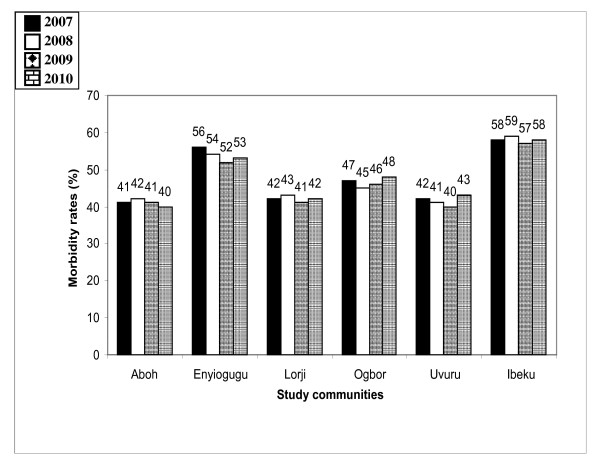
**Malaria morbidity rates as a proportion of total outpatients in health facilities in communities in Aboh LGA between 2007-2010**.

**Figure 3:**
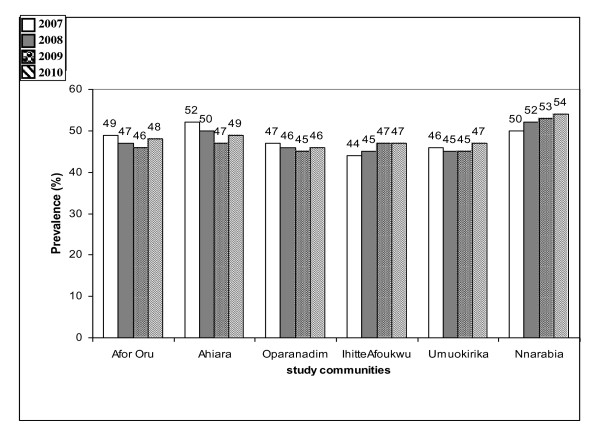
**Malaria morbidity rates as a proportion of total outpatients in health facilities in communities in Ahiazu LGA between 2007-2010**.

The most common malaria symptoms reported were headache, fever, chills and joint pains. Splenomegally, anemia and cerebral malaria were the most severe morbidity indicators reported. Out of 143, 528 out patients registered at health facilities during the period of study, about 30.2% of the cases were actually attributed to malaria. The annual mean malaria morbidity rate was 52.8% of total facility attendance. The pattern of mean malaria morbidity rate by health facility records in the 4 year period was not exactly similar among communities in the 3 LGAs (*p *> 0.05) (Figures 1, 2 and 3).

**Figure 4:**
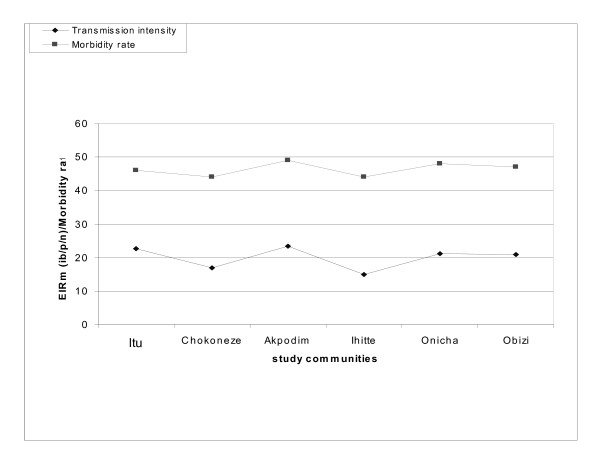
**Relationship between malaria transmission and morbidity rates in study communities in Ezinihitte LGA (2010)**.

**Figure 5:**
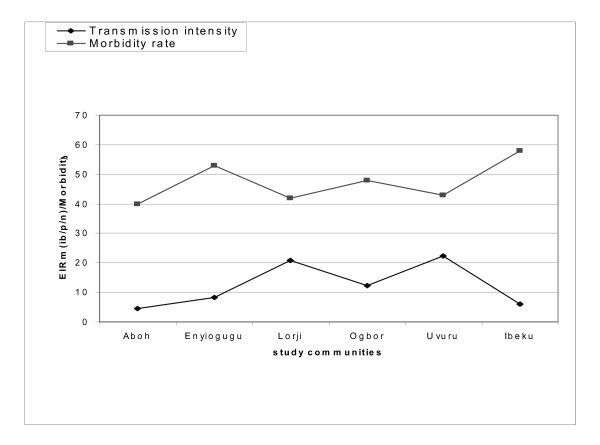
**Relationship between malaria transmission and morbidity rates in study communities in Aboh Mbaise LGA (2010)**.

**Figure 6:**
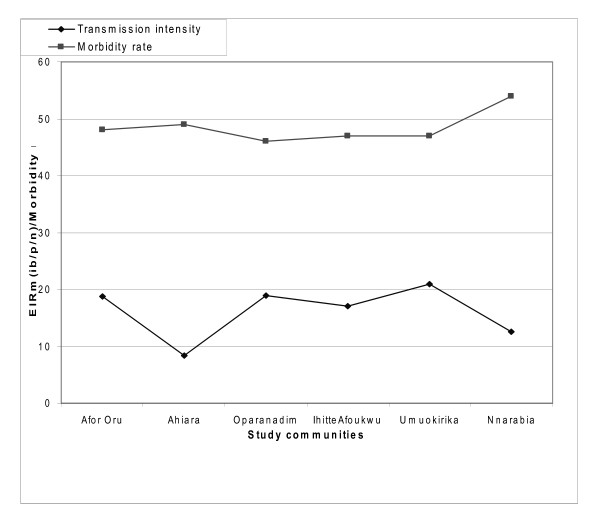
**Relationship between malaria transmission and morbidity rates in study communities in Ahiazu Mbaise LGA (2010)**.

The relationship between malaria transmission intensity and morbidity patterns in the study communities as at the period of study (2010) are represented in Figures 4, 5 and 6. There were no significant differences (*p *< 0.05) in the patterns of malaria transmission and morbidity rates among the communities

## Discussion

This study demonstrates that malaria transmission in the study area is high. The variation in malaria transmission intensity between the study communities was not very significant, although slightly higher mean entomological inoculation rates (EIRm) were observed in some communities located at lower altitudes. The proximity of such communities to rivers and river beds and the ability of the vector mosquitoes to easily reach human populations from their resting habitats may be implicated in this phenomenon [2,11]. The importance of altitude in malaria transmission have been documented [7]. As one ascends into higher altitudes, temperature gradually decreases rendering the weather conditions unfavorable for malaria transmission [11]. It is very important to look into the problems of malaria transmission in the study area and other parts of Southeastern Nigeria, taking into serious consideration the peculiar ecology, climatology and other factors and human activities which encourage mosquito breeding and malaria transmission. It is also important to note that these human activities directly affect the social, cultural and economic lives of the people.

Malaria morbidity rates as recorded in health facilities were relatively high compared with what is obtained in some other parts of Nigeria [6]. However, Similar observations have also been made in other parts of Africa[11,12]. This phenomenon is particularly disturbing in the present study and may be attributed to factors such as late acquisition of natural immunity, malnutrition or nutritional status and late recognition and treatment of malaria [13].

A significant association between transmission intensity and morbidity rates was found in this study. This is consistent with previous reports that high morbidity rates were found in communities with high malaria transmission in Tanzania [11] and that high entomological inoculation rates were associated with higher malaria morbidities in western Kenya [12]. This trend provides evidence for a likely benefit of interventions aimed at reducing transmission intensity for low and middle altitude areas [13]. However the reverse has been predicted for higher altitude communities were low transmission intensity tends to correspond with higher morbidity rates [14]. This may be explained by concerns that low transmission intensity by delaying acquisition of natural immunity against malaria may merely shift the burden of the disease to an older age group on subsequent exposure to transmission of the disease [14,15]. Furthermore, it has been observed that the degree of malaria immunity acquired by individuals living in endemic areas depend on the amount of exposure to infections and genetically determined immunological responses [16]. In areas of high stable transmission such as the study area, the incidence of the clinical malaria peaks is between 1 and 5 years of age and then declines rapidly as effective immune responses develop [14]. The epidemiological situation of malaria in the World remains a major threat to public health. In Africa, the global malaria eradication programme of the 1950s was not implemented due to high malaria endemicity, poor infrastructure and lack of financial resources. After the failure of the global eradication approach in 1992, WHO changed to a malaria control strategy based on early diagnosis and prompt treatment, implementation of selective, sustainable, preventive measures including vector control and strengthening of local capacities for assessment of malaria situation and its determinants in the affected countries. In 2004, the World Health Organization estimated the global incidence of malaria at 300-500 clinical cases annually, causing 1.5 to 2.7 million deaths each year. Today, more than 90% of malaria morbidity and mortality occur in Sub-Saharan Africa (SSA), where malaria accounts for an estimated 25% of all childhood mortality below the age of five. Recent studies suggest that this percentage might even be higher because of the contribution of malaria as an indirect cause of death [8].

## Conclusion

The epidemiological picture of malaria is worsening with the spread of *Plasmodium falciparum *resistance to existing first line drugs and vector resistance to insecticides. This therefore makes transmission reduction strategies very important. This study ascertained high malaria morbidity rates and severe malaria morbidity indicators to be relatively higher in communities located at higher altitudes with higher transmission intensities. This calls for scaling up of the insecticide treated bed nets strategy especially in high altitude rural areas. In addition more concerted effort should be made towards the production of more effective transmission blocking vaccines.

## Competing interests

The authors declare that they have no competing interests.

## Authors' contributions

UMC conceptualized, designed, carried out entomological and parasitic assays and also contributed in drafting the manuscript. INSD analyzed the hospital records, contributed in drafting the manuscript and also reviewed the manuscript. Both authors read and approved the final manuscript.
